# MICA/B and ULBP1 NKG2D ligands are independent predictors of good prognosis in cervical cancer

**DOI:** 10.1186/1471-2407-14-957

**Published:** 2014-12-15

**Authors:** Hanbyoul Cho, Joon-Yong Chung, Sunghoon Kim, Till Braunschweig, Tae Heung Kang, Jennie Kim, Eun Joo Chung, Stephen M Hewitt, Jae-Hoon Kim

**Affiliations:** Department of Obstetrics and Gynecology, Gangnam Severance Hospital, Yonsei University College of Medicine, 146-92 Dogok-Dong, Gangnam-Gu, Seoul, 135-720 Korea; Institute of Women’s Life Medical Science, Yonsei University College of Medicine, Seoul, Korea; Tissue Array Research Program, Laboratory of Pathology, National Cancer Institute, National Institutes of Health, 10 Center Drive, MSC 1500, Bethesda, MD 20892 USA; Department of Obstetrics and Gynecology, Severance Hospital, Yonsei University College of Medicine, Seoul, Korea; Institute of Pathology, RWTH Aachen University, Aachen, Germany; Department of Immunology, College of Medicine, Konkuk University, Chungju, Korea; Radiation Oncology Branch, Center for Cancer Research, National Cancer Institute, National Institutes of Health, Bethesda, MD USA

**Keywords:** Cervical cancer, Tissue microarray, Immunohistochemistry, NKG2D ligands

## Abstract

**Background:**

NKG2D (natural killer group 2, member D) is thought to play an important role in mediating the activation of anticancer immune response. Expression of NKG2D ligands (NKG2DLs) is pronounced in malignancies and the heterogeneity of NKG2DL expression remains unclear. Here, we investigate the expression and clinical significance of NKG2DLs in cervical cancer.

**Methods:**

Immunohistochemical analyses of MICA/B, ULBP1, ULBP2, ULBP3, RAET1E, and RAET1G were performed using tissue microarray analysis of 200 cervical cancers, 327 high-grade cervical intraepithelial neoplasias (CINs), 99 low-grade CINs, and 541 matched nonadjacent normal cervical epithelial tissues and compared the data with clinicopathologic variables, including the survival of cervical cancer patients.

**Results:**

MICA/B, ULBP1, and RAET1E expression was higher in cervical cancer than in low-grade CIN (*p* < 0.001, *p* = 0.012, *p* = 0.013, respectively) and normal cervix (all *p* < 0.001). Among these markers, expression of ULBP1 was significantly different depending on patient tumor stage (*p* = 0.010) and tumor size (*p* = 0.045). ULBP1 expression was correlated with MICA/B (*p* < 0.001) and ULBP2 (*p* = 0.002) expression in cervical cancer. While MICA/B+ or ULBP1+ patients had improved disease-free survival time (*p* = 0.027 and *p* = 0.009, respectively) relative to that of the low expression group, RAET1E+ or RAET1G+ was correlated with shorter survival time (*p* = 0.018 and *p* = 0.029, respectively). However, in terms of overall survival, the ULBP1+ group had significantly longer survival time than the low expression group (*p* = 0.009). Multivariate analysis indicated that MICA/B+/ULBP1+ (HR = 0.16, *p* = 0.015) and ULBP1+ (HR = 0.31, *p* = 0.024) are independent prognostic factors of disease-free survival in cervical cancer.

**Conclusions:**

High expression of either ULBP1 or MICA/B and ULBP1 combined is an indicator of good prognosis in cervical cancer, suggesting their potential utility as prognostic tests in clinical assessment.

## Background

Cervical cancer is the second most common malignant tumor affecting women worldwide, causing an estimated 273,200 deaths annually, and is the most common tumor in developing countries [1]. Persistent infection with one of the high-risk forms of human papillomavirus (HPV; types 16 and 18) has been shown to be a major etiological factor of HPV-related premalignant lesions and cervical cancer [2]. Although the vast majority of cervical cancers are derived from cervical intraepithelial neoplasia (CIN), the majority of genital HPV infections are clinically undetectable and clear in 10–16 months; only a very small proportion progress into an invasive cervical cancer. It is well known that persistent HPV infection causes progression from low-grade CIN to high-grade CIN, and eventually to a malignant cervical cancer in a multistep process [3–5]. The progression of these lesions may be due to an adverse tumor environment, wherein the mucosal immune response may be unable to completely remove malignant cells.

Host immune response to HPV appears to be critical in determining the outcome of infection. For example, among immunocompromised women, HPV infection is detected more frequently, the incidence of CIN is higher, and the risk of CIN recurrence after treatment is higher [6]. Innate immune response is thought to be the first line of defense at mucosal surfaces. Natural killer (NK) cells are important cytolytic and cytokine-producing effector cells of the innate immune system. These cells have the ability to attack tumor cells and cells infected with viruses and some bacteria without presentation of tumor-specific antigens [7]. Furthermore, intratumoral NK cell accumulation has been correlated with improved survival rates in patients with various solid tumors [8, 9]. In the case of cervical cancer, Garzetti *et al.* reported that NK cell activity was related to prognostic parameters and clinical outcome [10].

NKG2D (natural killer group 2, member D) is a C-type lectin-like activating receptor expressed on the surface of NK cells and a variety of T cell subsets including CD8+ cytotoxic T cells [11]. Human NKG2D ligands (NKG2DLs) consist of two members of the MHC class I-related chain (MIC) family (MICA and MICB) and six members of the UL16 binding protein or retinoic acid early transcript (ULBP/RAET) family (ULBP1, ULBP2, ULBP3, RAET1E, RAET1G, and RAET1L) [12]. NKG2DL expression is highly restricted in healthy tissues, but can be stimulated by multiple stimuli, including infection and heat shock, and by cellular transformation [12]. It is also broadly expressed in a variety of tumors, including hematologic and epithelial malignancies [12, 13], cervical cancers [14], and cancer cell lines [15, 16]. The mechanisms regulating NKG2DL expression in carcinogenesis still remain to be elucidated, although activation of DNA damage response pathways and expression of the BCR/ABL oncogene have been implicated [16–18].

In the current study, we hypothesized that the expression of some or all types of NKG2DLs in cervical neoplasias are correlated with tumor progression. To explore this hypothesis, we investigated the expression of MICA/B, ULBP1, ULBP2, ULBP3, RAET1E, and RAET1G in normal cervical epithelium and cervical neoplasia in a large series of formalin-fixed, paraffin-embedded tumor samples made by using high-throughput tissue microarray (TMA) technology. Because HPV is associated with cervical carcinogenesis, we also examined the relationship between HPV status and NKG2DL expression in cervical neoplasia.

## Methods

### Patients and tumor samples

The study subjects were comprised of 200 cervical cancer and 426 cervical intraepithelial neoplasias (CINs) patients who underwent surgical resection at Gangnam Severance Hospital, Yonsei University College of Medicine between March 1996 and March 2010. Additional paraffin blocks were provided by the Korea Gynecologic Cancer Bank through the Bio & Medical Technology Development Program of the Ministry of Education, Science and Technology, Korea. All patients had a histological diagnosis of cervical carcinoma or CIN, and the cervical cancer patients were clinically staged according to the International Federation of Gynecology and Obstetrics (FIGO) staging system. Patients with cervical cancer underwent type 3 radical hysterectomy with pelvic lymph node dissection, and, in cases of increased risk of relapse (assessed from spread to lymph node, parametrial invasion, and cancer close to resection margins), platinum-based concurrent chemoradiation was added. Medical records were reviewed to obtain data including age, Hybrid Capture^®^ 2 results, surgical procedure, survival time, and survival status. Response to therapy was assessed by either computed tomography or magnetic resonance imaging in accordance with the Response Evaluation Criteria in Solid Tumors (RECIST, version 1.0) [17]. Data on tumor size, cell type, tumor grade, and lymph node metastases were obtained from pathology reports. Tissue samples were collected from patients who had signed informed consent forms, which was approved by the Institutional Review Boards of Gangnam Severance Hospital. This study was additionally approved by the Office of Human Subjects Research at the National Institute of Health.

### Tissue microarray construction

Tissue cores, from formalin-fixed, paraffin-embedded tissue blocks obtained from 626 patients with primary invasive cervical cancer or CIN and 541 matched nonadjacent normal cervical epithelia were arrayed into a recipient paraffin block with a manual tissue arrayer MTA-1 (Beecher Instruments Inc., Silver Spring, MD). These normal cervical tissue cores were obtained from the same block at locations distant from the cancer cells or from different block that contained enough normal epithelial cells for further IHC analyses. For each case, a representative tumor area was carefully selected from a hematoxylin and eosin (H&E)-stained section of the donor block. Four 1.0-mm-diameter cores consisting of matched tumor specimen and normal epithelial samples were retrieved from selected regions of a patient’s donor block. The presence of tumor tissues on the tissue microarray (TMA) was verified with H&E staining. At every 50th section, multiple 5-μm-thick sections were cut with a microtome and H&E staining of TMA slides were examined for the presence of tumor cells.

### Cell culture

Human cervical cancer cell lines were obtained from two sources: C-33A, CaSki, HeLa, ME-180 and SiHa from American Type Culture Collection (ATCC, Manassas, VA) and SUN-17 from Korean Cell Line Bank (KCLB, Seoul, Korea). CaSki, HeLa, ME-180, and SUN-17 cells were cultured *in vitro* in RPMI 1640 while C-33A and SiHa cells were cultured in DMEM (Dulbecco’s modified Eagle’s medium). Both media were supplemented with 10% fetal bovine serum, 50 units/ml of penicillin/streptomycin, 2 mM L-glutamine, 1 mM sodium pyruvate, and 2 mM non-essential amino acids, and cells were grown at 37°C with 5% CO_2_.

### Flow cytometry analysis

For *in vitro* flow cytometry analysis, 2 × l0^5^ tumor cells were incubated with 0.5 μg recombinant human NKG2D Fc chimera (R & D systems, Minneapolis, MN) and then PE-conjugated anti-human Fc secondary antibody was used as a detection antibody (BD Bioscience, San Jose, CA). CELLQuest software (Becton Dickinson Immunocytometry System, Mountain View, CA) was used for FACScan analysis.

### Immunohistochemistry

Immunohistochemical staining of MICA/MICB, ULBP1, ULBP2, ULBP3, RAET1E, and RAET1G was performed by using streptavidin-biotin peroxidase method. Prior to applying IHC with TMA section, we tested whole section for immunohistochemial staining condition. Briefly, the TMA sections were deparaffinized by xylene and then rehydrated through a descending alcohol gradient. Endogenous peroxidase activity was blocked by 10 min of incubation in 3% H_2_O_2_. To retrieve antigenicity, sections were immersed in antigen retrieval buffer, pH 9 (Dako, Carpinteria, CA), and heated for 20 min in a steam pressure cooker (Pascal, Dako). Slides for ULBP1 and ULBP2, however, were heated for 10 min instead of 20 min at high pressure. To block nonspecific staining, sections were treated with Protein Block (Dako) for 20 min. The sections were incubated with anti-MICA/MICB antibody (Novus Biologicals, Littleton, CO; mouse monoclonal antibody, Clone 6D4, 1:50 for 120 min), anti-ULBP1 antibody (Sigma-Aldrich, St. Louis, MO; rabbit polyclonal antibody, Cat.# HPA007547, 1:50 for 120 min), anti-ULBP2 antibody (R&D systems; goat polyclonal antibody, Cat.# AF1298, 1:50 for 30 min), anti-ULBP3 antibody (Abcam, Cambridge, MA; mouse monoclonal antibody, Clone MM0594-6E12, 1:500 for 120 min), anti-RAET1E antibody (Novus Biologicals; mouse polyclonal antibody, Cat.# H00135250-B01, 1:2000 overnight at 4°C), and anti-RAET1G antibody (Novus Biologicals; mouse polyclonal antibody, Cat.# H00353091-B01, 1:1000 overnight at 4°C) in a Dako autostainer plus (Dako). Different systems were used for the detection of primary antibodies: Dako EnVision + Dual Link System-HRP (Dako) for MICA/MICB and ULBP3; Dako EnVision FLEX+ (Dako) for ULBP1, RAET1E, and RAET1G; and Dako LSAB^®^2 System-HRP (Dako) for ULBP2. The stain was visualized using DAB^+^ kit (3,3’-Diaminobenzidine; Dako) and then lightly counterstained with hematoxylin. The slides were covered and observed under a light microscope (Axioplot, Carl Zeiss, Jena, Germany). Negative controls were processed by omitting the primary antibodies, and TMAs included colorectal cancer positive control tissues [19].

### Evaluation of IHC staining

For the assessment of NKG2DL staining, two scores were assigned to each core: (a) the staining intensity (no evidence of staining, 0; weak staining, 1+; moderate staining, 2+; and strong positive staining in most cells, 3+) and (b) the percentage of positively stained epithelial cells (no cells staining positive, 0; less than 25% of cells staining positive, 1+; 25–50% of cells staining positive, 2+; 50–75% of cells staining positive, 3+; and more than 75% of cells staining positive, 4+). An overall protein expression score was calculated by multiplying the intensity and positivity scores (overall score range, 0–12). The IHC staining score was then dichotomized into low expression (≤ mean score of cancer specimens) and high expression (> mean score of cancer specimens). Slides were scored without any clinical information, and the final immunostaining score reported was the average of two independent pathologists, both with experience in the analysis of tissue microarray.

### Statistical analysis

Statistical analyses were performed using SPSS version 18.0 (SPSS Inc., Chicago, IL). The statistical significance of the differences in staining score of NKG2DLs in the different groups was calculated by the Mann–Whitney test and the Kruskal-Wallis test. Overall and disease-free survival curves were generated by the Kaplan-Meier method and the difference between the survival curves was calculated by the log-rank test. The Cox proportional hazards model was used for multivariate analysis to determine independent significance of relevant clinical covariates. In all cases, a *p* value < 0.05 was considered statistically significant.

## Results

### Clinicopathologic characteristics of patient cohort

Table 1 summarizes patient clinicopathologic characteristics. The overall mean age of patients was 40.6 ± 9.8 years for low-grade CIN, 38.9 ± 11.2 years for high-grade CIN, and 49.4 ± 11.7 years for cervical cancer. The distribution of FIGO staging for the 200 cases of cervical cancer is as follows: 138 stage I, 53 stage II, and 9 stage IV. The following cell types were assigned according to World Health Organization (WHO) criteria: 164 squamous cell carcinomas, 30 adenocarcinomas/adenosquamous carcinomas, 4 small cell carcinomas, 1 neuroendocrine, and 1 clear cell carcinomas. HC2-confirmed HPV infection rate was 78.7% (74/94) in low-grade CIN, 92.2% (226/245) in high-grade CIN, and 93.9% (92/98) in cervical cancer.

**Table 1:** **Characteristics of patients**

Variable	Number	%
**Age (years)**	42.58 ± 12.15^a^	
**Diagnostic category**		
Normal	541	46.4
Low-grade CIN	99	8.5
High-grade CIN	327	28.0
Cancer	200	17.1
**FIGO stage**		
I	138	69.0
II	53	26.5
IV	9	4.5
**Tumor grade** ^b^		
Well/Moderate	117	65.7
Poor	61	34.3
**Cell type**		
SCC	164	82.0
AC/ASC	30	15.0
Others	6	3.0
**Tumor size**		
≤ 4 cm	145	72.5
> 4 cm	55	27.5
**Lymphovascular invasion** ^**c**^		
Negative	95	64.6
Positive	52	35.4
**Lymph node metastasis** ^d^		
Negative	141	77.5
Positive	41	22.5
**Chemoradiation response** ^e^		
Good	38	77.6
Bad	11	22.4
**SCC antigen** ^**f**^		
Negative	100	66.7
Positive	50	33.3
**HPV test in CIN** ^**g**^		
Negative	39	11.5
Positive	300	88.5

CIN, cervical intraepithelial neoplasia; FIGO, International Federation of Gynecology and Obstetrics; SCC, squamous cell carcinoma; AC, adenocarcinoma; ASC, adenosquamous carcinoma. ^a^mean ± standard deviation, ^b^calculated only for the 178 cases with available information on tumor grade, ^c^calculated only for the 147 cases with available information on examined lymphovascular invasion, ^d^calculated only for the 182 cases with available information on examined lymph nodes, ^e^calculated only for the 49 cases with available information on chemoradiation response, ^f^calculated only for the 114 cases with available information on SCC antigen level, ^g^calculated only for the 339 CIN cases with available information on HPV infection.

### Confirmation of NKG2DLs in cervical cancer cell lines

**Figure 1:**
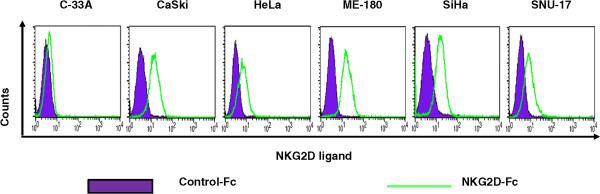
**Characterization of the binding of various human cervical cancer cell lines with human NKG2D Fc chimera.** Various human cervical cancer cells were incubated with recombinant human NKG2D chimera and then anti-Fc-PE. Characterization was performed via flow cytometry. As a control, each kind of cell was stained with anti-Fc-PE antibodies.

Expression of NKG2DLs was determined by flow cytometric assay using recombinant human NKG2D-Fc chimera protein in cell cultures prior to IHC analysis for individual NKG2DL expression in cervical cancer tissues. NKG2D-Fc chimera protein binds to NKG2D through ligand-receptor interaction on the cell surface, and it is detected by anti-Fc antibody conjugated with fluorophores. As a result, using NKG2D-Fc chimera protein in flow cytometric assay allows us to confirm the expression and binding ability of NKG2DLs even without a characterization of ligands. As shown in Figure 1, expression of NKG2DLs was determined in six different cervical cancer cell lines (C-33A, CaSki, HeLa, ME-180, SiHa and SUN-17). Five cancer cell lines (all but C-33A) expressed NKG2DLs, which could bind to NKG2D, supporting the hypothesis that cervical cancers express NKG2DLs *in vivo*.

### Expression of individual NKG2DLs in cervical neoplasias

**Figure 2:**
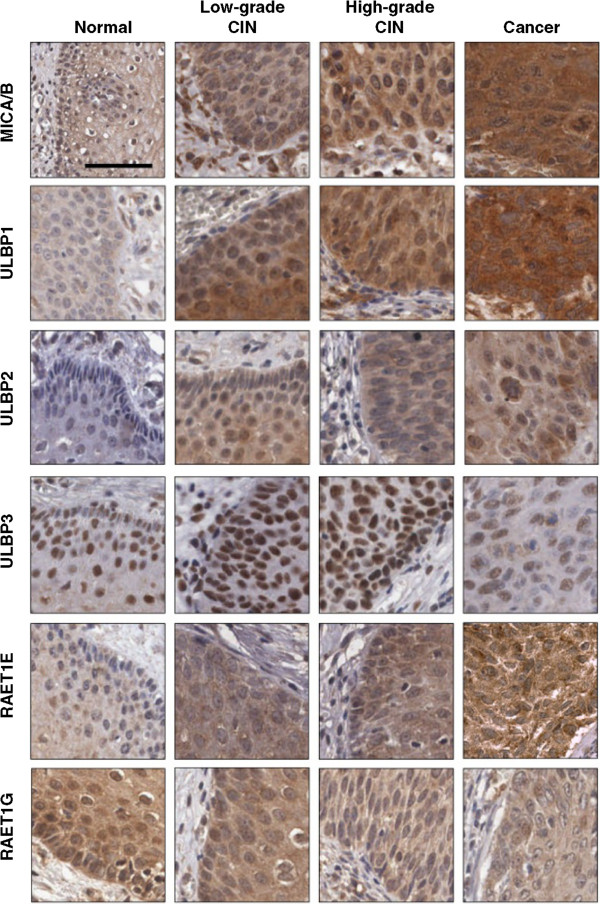
**Immunohistochemical stains for MICA/MICB, ULBP1, ULBP2, ULBP3, RAET1E, and RAET1G on tissue microarrays.** All photos are at the same magnification. Bar: 50 μm.

We then performed IHC analysis of MICA/MICB, ULBP1, ULBP2, ULBP3, RAET1E, and RAET1G in 200 cervical cancer specimens, 327 high-grade CINs, 99 low-grade CINs, and 541 matched nonadjacent normal cervical epithelial tissue samples. Representative immunohistochemical expression of individual NKG2DLs are presented in Figure 2. ULBP3 was expressed exclusively in the nucleus in both tumor and normal epithelial cells, while the other markers were expressed primarily in the cytoplasm, with some cases also demonstrating weak nucleus staining (Figure 2). Scoring results from the IHC analyses are summarized in Table 2. The TMA contains 200 cases of cervical cancer, however due to the complexity of sectioning and staining, between 180 and 195 samples could be interpreted for the individual marker. Invasive cervical cancer tissues had higher MICA/B, ULBP1, and RAET1E expression than CIN or normal cervical epithelial tissues (all *p* < 0.001). This trend of progressively increasing MICA/B, ULBP1, and RAET1E expression corresponded to the phases of cervical cancer progression and was significant according to Spearman’s rank correlation (*ρ*-value of 0.313 [*p* < 0.001], 0.285 [*p* < 0.001], or 0.136 [*p* < 0.001], respectively). ULBP2 and ULBP3 expression, on the other hand, was higher in low-grade CIN than in normal epithelium but gradually decreased in high-grade CIN and cervical cancer (*p* = 0.001 and *p* = 0.017, respectively).

**Table 2:** **IHC scores of NKG2DLs MICA/B, ULBP1, ULBP2, ULBP3, RAET1E, and RAET1G in 1,167 TMA specimens**

		Number	%	Mean score	95% CI		***p***value
					Lower	Upper	
MICA/B	Normal	457	45.2	4.83	4.62	5.04	<0.001
	Low-grade CIN	90	8.9	4.89	4.53	5.25	
	High-grade CIN	270	26.7	6.30	6.00	6.61	
	Cancer	195	19.3	6.78	6.43	7.13	
	Total	1012	100.0	5.60	5.45	5.76	
ULBP1	Normal	500	45.9	4.82	4.64	5.00	<0.001
	Low-grade CIN	92	8.4	5.54	5.14	5.94	
	High-grade CIN	314	28.8	6.05	5.78	6.32	
	Cancer	183	16.8	6.66	6.27	7.04	
	Total	1089	100.0	5.54	5.40	5.68	
ULBP2	Normal	458	43.5	3.47	3.30	3.65	0.001
	Low-grade CIN	93	8.8	4.23	3.82	4.63	
	High-grade CIN	318	30.2	3.94	3.73	4.16	
	Cancer	184	17.5	3.71	3.40	4.02	
	Total	1053	100.0	3.72	3.60	3.84	
ULBP3	Normal	455	44.0	5.22	5.02	5.41	0.017
	Low-grade CIN	93	9.0	5.84	5.37	6.31	
	High-grade CIN	307	29.7	5.34	5.05	5.63	
	Cancer	180	17.4	4.91	4.56	5.26	
	Total	1035	100.0	5.26	5.11	5.40	
RAET1E	Normal	461	44.4	3.57	3.47	3.68	<0.001
	Low-grade CIN	94	9.1	3.77	3.54	3.99	
	High-grade CIN	302	29.1	3.76	3.62	3.91	
	Cancer	181	17.4	4.38	4.12	4.65	
	Total	1038	100.0	3.79	3.71	3.87	
RAET1G	Normal	470	44.2	5.11	4.95	5.27	0.001
	Low-grade CIN	95	8.9	5.17	4.83	5.50	
	High-grade CIN	314	29.5	4.61	4.39	4.82	
	Cancer	184	17.3	5.11	4.78	5.45	
	Total	1063	100.0	4.97	4.85	5.08	

### Association of NKG2DLs

To determine the association between expression of MICA/MICB, ULBP1, ULBP2, ULBP3, RAET1E, and RAET1G, Spearman’s rank correlation analysis was performed (Table 3). Cervical cancer lesions were evaluated for co-expression between individual NKG2DLs. MICA/B expression was significantly correlated with the expression of ULBP1 (Spearman’s rho = 0.677, *p* < 0.001) or ULBP2 (Spearman’s rho = 0.257, *p* < 0.001), whereas MICA/B expression was not significantly correlated with those of ULBP3 (Spearman’s rho = 0.119, *p* = 0.112), RAET1E (Spearman’s rho = 0.044, *p* = 0.555), or RAET1G (Spearman’s rho = -0.042, *p* = 0.574).

**Table 3:** **Spearman’s rank correlation coefficient in cervical cancer specimens**

			MICA/B	ULBP1	ULBP2	ULBP3	RAET1E	RAET1G
Spearman’s correlation	MICA/B	*ρ*	1.000	.677	.257	.119	.044	-.042
		*p*	NA	< .001	< .001	.112	.555	.574
		*n*	195	183	181	179	181	182
	ULBP1	*ρ*	.677	1.000	.232	.140	.018	-.055
		*p*	< .001	NA	.002	.068	.820	.476
		*n*	183	183	174	171	169	170
	ULBP2	*ρ*	.257	.232	1.000	.333	-.119	-.107
		*p*	< .001	.002	NA	< .001	.121	.162
		*n*	181	174	184	176	172	174
	ULBP3	*ρ*	.119	.140	.333	1.000	.268	.158
		*p*	.112	.068	< .001	NA	< .001	.038
		*n*	179	171	176	180	172	173
	RAET1E	*ρ*	.044	.018	-.119	.268	1.000	.616
		*p*	.555	.820	.121	< .001	NA	< .001
		*n*	181	169	172	172	182	179
	RAET1G	*ρ*	-.042	-.055	-.107	.158	.616	1.000
		*p*	.574	.476	.162	.038	< .001	NA
		*n*	182	170	174	173	179	184

NA, not applicable.

### Prognostic significance of NKG2DL expression

**Figure 3:**
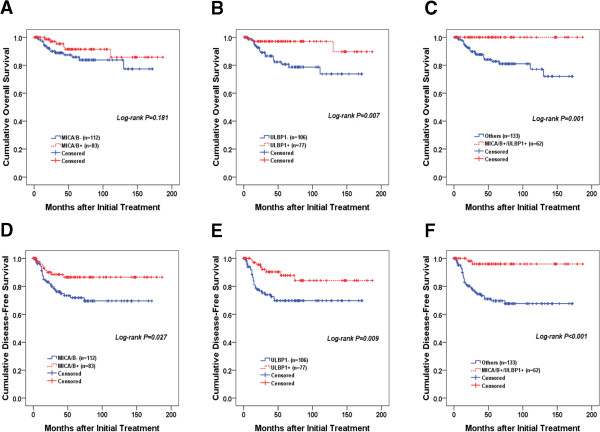
**Kaplan–Meier plots for overall (A, B, C) and disease-free (D, E, F) survival for patients categorized by MICA/B or ULBP1 expression.**

Finally, to investigate the prognostic significance of the expression of individual NKG2DLs in cervical cancer, we studied the correlation of NKG2DL expression with overall and disease-free survival. Clinicopathologic and outcome information were available for all 200 cervical cancer patients who were monitored for survival and recurrence. Kaplan-Meier plots demonstrated that patients with high MICA/B (Log-rank *p* = 0.027) or ULBP1 (Log-rank *p* = 0.009) expression and low RAET1E (Log-rank *p* = 0.018) or RAET1G (Log-rank *p* = 0.029) expression had significantly longer disease-free survival time (Table 4, Figure 3A and B). When analyzed individually for effect on overall survival, high ULBP1 expression predicted significantly longer survival time (Log-rank *p* = 0.007) (Table 4, Figure 3D and E). In particular, when survival of patients with expression of high MICA/B/high ULBP1 was compared with those of other patients, Kaplan-Meier analysis revealed a significant difference in both disease-free (*p* < 0.001, Figure 3C) and overall survival (*p* = 0.001, Figure 3F). A Cox multivariate proportional hazards analysis showed that advanced stage (hazard ratio = 3.60 [95% CI, 1.36–9.55], *P* = 0.010) and lymph node metastasis (hazard ratio = 2.71 [95% CI, 1.08–6.79], *p* = 0.032) were related to poor disease-free survival, whereas high ULBP1 (hazard ratio = 0.31 [95% CI, 0.11–0.86], *p =* 0.024) and high MICA/B/high ULBP1 (hazard ratio = 0.16 [95% CI, 0.13–0.70], *p =* 0.015) expression was related to good disease-free survival (Table 2). When analyzed for effect on overall survival, advanced stage (hazard ratio = 2.77 [95% CI, 1.13–7.76], *p* = 0.025) and high ULBP1 (hazard ratio = 0.27 [95% CI, 0.07–0.97], *p =* 0.044) expression were significant independent prognostic factors in multivariate analysis.

**Table 4:** **Overall and disease-free survival analysis of NKG2DLs MICA/B, ULBP1, ULBP2, ULBP3, RAET1E, and RAET1G**

	Disease-Free Survival Time			Overall Survival Time		
	Mean	95% CI	Log-rank ***P***	Mean	95% CI	Log-rank ***P***
MICA/B						
Low (*n* = 112)	127	112-141	0.027	146	133-158	0.181
High (*n* = 83)	164	150-178		169	155-183	
ULBP1						
Low (*n* = 106)	125	110-140	0.009	139	125-152	0.007
High (*n* = 77)	163	148-178		177	167-188	
ULBP2						
Low (*n* = 93)	144	127-160	0.279	165	152-178	0.640
High (*n* = 91)	148	134-163		155	141-169	
ULBP3						
Low (*n* = 79)	136	121-151	0.364	147	134-160	0.707
High (*n* = 101)	139	122-157		159	144-173	
RAET1E						
Low (*n* = 123)	157	144-170	0.018	163	151-175	0.935
High (*n* = 59)	116	96-137		149	135-164	
RAET1G						
Low (*n* = 104)	159	146-172	0.029	166	154-179	0.234
High (*n* = 80)	127	108-146		149	132-165	

IHC staining scores of the NKG2DLs were dichotomized into low expression (≤ mean score of cancer specimens) and high expression (> mean score of cancer specimens).

## Discussion

Studies based on various in vivo models suggest that the immune system not only protects the host from early-stage tumors, but also promotes tumor growth through a process described as immunoediting, immunosculpting or cancer immune system, which can result in the outgrowth of more aggressive tumor cells via exposure to immune effectors and loss of immunogenicity [18, 20, 21]. In addition, these in vivo cancer models also strongly suggest that the activating immune receptor NKG2D stimulates anti-cancer immune responses [22–24]. Considering the fact that many human primary tumors and tumor-derived cell lines express NKG2DLs [12, 16], a great deal of research is currently focused on investigating the role of NKG2D in host-mediated tumor immunity. Despite recent progress, the biological functions of NKG2DLs are not yet fully understood, and the clinicopathologic significance of these ligands in cervical cancer has yet to be reported.

In the present study, we investigated NKG2DL expression and further explored the clinical significance of NKG2DLs by using samples from a large cohort of patients including cervical cancer, precursor and corresponding normal specimens. Immunohistochemistry analysis revealed that MICA/B and ULBP1 were significantly upregulated in cervical cancer tissues compared to their corresponding normal tissues. Notably, higher MICA/B and ULBP1 expression correlated with more advanced stages of cervical carcinogenesis, increasing from normal cervical tissue to progressively advanced stages of cervical cancer precursors (low-grade and high-grade CIN) and ultimately to invasive cancer. These findings suggest that MICA/B and ULBP1 upregulation follows malignant transformation in cervical carcinogenesis. Thus, not only can these proteins be used as potential markers in treatment and surveillance of cervical cancer, but they may also have some utility in screening. Since their expression increases even in early stages of cervical cancer, MICA/B and ULBP1 expression can be used to identify precursor lesions (i.e., CIN) that are at high risk of developing invasive cervical cancer.

In addition to the correlations between NKG2DL expression levels and stages of cervical carcinogenesis, NKG2DL expression was heterogeneous in primary cancers, as not all of the ligands were highly expressed in the same tumor. More specifically, MICA/B, ULBP1 and RAET1E expression was significantly increased in cervical cancer tissues, while ULBP2 and ULBP3 expression were higher in low-grade CIN tissues, but lower in high-grade CIN and cervical cancer tissues. Similar to our study, Textor et al. demonstrated that NKG2DL MICA expression was upregulated in squamous cervical carcinoma tissues (*n* = 15) compared to CIN (*n* = 28) and normal ectocervical tissues (*n* = 10) while ULBP2 was strongly expressed in normal ectocervical tissues [14]. Although the mechanism for this heterogeneity in NKG2DL expression is complex and unclear at this time, it may be partially explained by the following reasons. First, this heterogeneity may be due to NKG2DLs having different promoters and NKG2D’s ability to be expressed independently in response to diverse stress response pathways. Secondly, evidence of post-transcriptional regulation of NKG2DLs, including the involvement of micro-RNAs (miR-20a, miR-93, miR106b, miR-302d, miR-372, miR-373 and miR-520d), also indicates that NKG2D expression is further differentially regulated [25]. Lastly, some stimuli, such as DNA damage response, have been reported to result in expression of all NKG2DLs tested, while other stimuli have been shown to induce expression of other specific NKG2DLs [26]. BCR/ABL, for example, regulates MICA, but not ULBP1-2 in K562 cells, while histone deacetylase inhibitor induces MICA, but not ULBP1-3 in hepatoma cells [27, 28].

Regardless of NKG2DL heterogeneity in expression, our prognostic model indicates that the presence of NKG2DLs is crucial for stimulating immune responses against tumors. Our survival analysis indicates that the high expression of NKG2DLs predicts a good prognosis, with the combination of ULBP1 and MICA/B predicting disease-free survival and ULBP1 independently predicting both disease-free and overall survival with statistical significance (Table 5). This is in agreement with a recent report on MIC/ULBP/RAET expression in 462 primary colorectal tumors, which states that high expression of MIC or RAET1G predicts improved patient survival [19]. Considering the fact that NKG2DLs are involved in anti-cancer immune responses, an association between high expression of NKG2DLs and good prognosis is quite reasonable. Thus, when identifying patients with cervical cancer at increased risk of tumor invasion and/or progression, examining the expression levels of ULBP1 or MICA/B via IHC may have utility. Furthermore, these findings all emphasize that NKG2DLs have important functions in the biological mechanism underlying the development and/or growth of human cervical cancer.

**Table 5:** **Cox proportional univariate and multivariate analysis of the association between prognostic variables and overall and disease-free survival in cervical cancer**

	Disease-free Survival hazard ratio [95% CI], ***P***value		Overall Survival hazard ratio [95% CI], ***P***value	
	Univariate analysis	Multivariate analysis	Univariate analysis	Multivariate analysis
**FIGO stage**	5.32 [2.75-10.30], <0.001	3.60 [1.36-9.55], 0.010	3.97 [1.65-9.54], 0.002	2.77 [1.13-7.76], 0.025
**Tumor grade (poor)**	1.65 [0.85-3.22], 0.137	NA	2.15 [0.89-5.21], 0.087	NA
**Tumor size (>4 cm)**	2.13 [1.09-4.14], 0.025	1.16 [0.47-2.90], 0.737	1.90 [0.78-4.65], 0.157	NA
**LN metastasis**	4.53 [2.14-9.61], <0.001	2.71 [1.08-6.79], 0.032	2.25 [0.77-6.59], 0.136	NA
**HPV+**	0.68 [0.08-5.21], 0.710	NA	0.38 [0.048-3.10], 0.370	NA
**MICA/B+**	0.43 [0.20-0.92], 0.032	0.54 [0.20-1.41], 0.213	0.53 [0.20-1.36], 0.189	NA
**ULBP1+**	0.36 [0.16-0.80], 0.012	0.31 [0.11-0.86], 0.024	0.21 [0.06-0.73], 0.014	0.27 [0.07-0.97], 0.044
**RAET1E+**	2.17 [1.12-4.2], 0.021	1.90 [0.84-4.32], 0.123	1.03 [0.41-2.60], 0.935	NA
**RAET1G+**	2.09 [1.05-4.15], 0.034	1.62 [0.68-3.87], 0.275	1.69 [0.70-4.09], 0.240	NA
**MICA/B+/ULBP1+**	0.11 [0.02-0.45], 0.002	0.16 [0.03-0.70], 0.015	0.026 [0.00-1.17], 0.060	NA

IHC staining scores of NKG2DLs were dichotomized into low (-) expression (≤ mean score of cancer specimens) and high (+) expression (> mean score of cancer specimens).

FIGO, International Federation of Gynecology and Obstetrics; LN, lymph node; HPV, human papilloma virus; CI, confidence interval; NA, not applicable.

However, in contrast to our results, recent studies by Li et al. demonstrated that high expression of MICA/B and ULBP2 is associated with poor prognosis in ovarian cancer patients [29]. This, together with our results, indicates that although NKG2DLs induce anti-cancer immune responses, the extent of these responses can markedly differ depending on the type of cancer. Such differences arise because NKG2DLs depend heavily on the tissue microenvironment where an assemblage of interactions by signals of cellular receptors and cytokines take place [30]. Since these interactions differ depending on the type of the cancer, different NKG2D responses are elicited [31, 32]. Thus, not only are NKG2DLs expressed in response to different cancer-related pathways, but also their expression is highly heterogeneous.

Intriguingly, although the results of our multivariate analysis were not significant, in univariate analysis, high RAET1E or RAET1G expression was associated with poor disease-free survival (Table 5). This finding agrees partially with a recent report on NKG2DL expression in 357 ovarian cancers, wherein high expression levels of ULBP2 and RAET1G was inversely correlated to the disease survival [33]. The lack of complete agreement may be due to the use of a different cancer type. However, more importantly, it can be explained by a study performed by Cao et al., in which RAET1E was able to produce soluble protein lacking a transmembrane region that weakened NKG2D-mediated NK cell cytotoxicity to tumor cells, despite that RAET1E and RAET1G contain both transmembrane and cytoplasmic domains [34]. Given the results of Cao et al.’s study and the fact that many isoforms were also stained during RAET1E and RAET1G IHC staining, it is possible that high RAET1E and RAET1G expression may be associated with poor prognosis.

## Conclusions

Overall, the expression of several NKG2DLs, namely MICA/B, ULBP1, and RAET1E, were increased in cervical cancer patients. In multivariate analysis, FIGO stage, lymph node metastasis, high ULBP1 expression and high combined MICA/B and ULBP1 expression were independent predictors of good prognosis. These findings all underscore the importance of NKG2D function in cervical tumor progression and cancer immunosurveillance, and suggest that combinatorial analysis of NKG2DL expression may assist in realizing improved prognostic classification of cervical and other carcinomas.

## Abbreviations

NKG2DL: NKG2D ligand
HPV: Human papillomavirus
CIN: Cervical intraepithelial neoplasia
LSIL: Low-grade squamous intraepithelial lesions
HSIL: High-grade squamous intraepithelial lesions
NK: Natural killer
FIGO: International federation of gynecology and obstetrics
TMA: Tissue microarray
H&E: Hematoxylin and eosin
SCC: Squamous cell carcinoma
AC: Adenocarcinoma
ASC: Adenosquamous carcinoma.
